# Justice Rendered

**Published:** 1878-07

**Authors:** 


Justice Rendered.—In our April number of the Journal, in the first article under the head of "Selections," injustice has been done the author and journal from which the article was copied, through oversight.

The article, "The Pathology of Seborrhœa," was written by Arthur VanHarlingen, M.D., chief of the skin clinic, Hospital of the University of Pennsylvania, and published in the "Archives of Dermatology," April, 1878.

Through what would appear a very careless oversight, the article was credited to the *Canada Medical and Surgical Journal*, and without giving the author's name.

